# Complete Genome Sequences of Two *Listeria monocytogenes* Serovars, 1/2a and 4b, Isolated from Dairy Products in Brazil

**DOI:** 10.1128/genomeA.01494-15

**Published:** 2015-12-17

**Authors:** Luiza Pieta, Fabrício Souza Campos, Roberta Fogliatto Mariot, Janira Prichula, Tiane Martin de Moura, Ana Paula Guedes Frazzon, Jeverson Frazzon

**Affiliations:** a Food Science Institute, Federal University of Rio Grande do Sul (UFRGS), Porto Alegre, Brazil; b Basic Health Sciences Institute, UFRGS, Porto Alegre, Brazil

## Abstract

*Listeria monocytogenes* is the foodborne pathogen responsible for a bacterial infection called listeriosis. Here, we present the whole-genome sequences of two *L. monocytogenes* serovars, 1/2a and 4b, which are considered the most prevalent in food processing plants and listeriosis outbreaks, respectively.

## GENOME ANNOUNCEMENT

*Listeria monocytogenes* is a psychrotrophic microorganism, widely distributed in the environment. Thirteen different serovars were already described for this bacterium, but three serovars denominated 1/2a, 1/2 b, and 4 b are responsible for most human cases of listeriosis (1). Four evolutive lineages (I, II, III, and IV) have been described for the microorganism, and serovar 1/2a belongs to lineage II, most involved with food contamination (2), as it is frequently isolated from food sources or food processing plants (3). Moreover, the serotype 4b is more related to outbreaks of the disease (37 to 64% of cases), and in addition to the frequent contamination of processed “ready-to-eat foods,” meats and cheeses, which require storage at low temperatures, the microorganism can also be found in many raw foods, especially vegetables, milk and fish (4, 5).

In this study, we report the complete genome sequences of two *L. monocytogenes* strain serovars, 1/2a and 4b, isolated from dairy products in Brazil, which had some genes related to biofilm formation, stress-response, and virulence tested by reverse-transcription real-time PCR (RT-qPCR) when growing at different temperatures (6).

The library of *L. monocytogenes* genomic DNA was prepared using the Nextera DNA library preparation kit 24 samples (Illumina, San Diego, CA, USA; Cat. #FC-121-1030), and the paired-end sequencing was performed on the Illumina MiSeq Platform (Illumina, San Diego, CA, USA) using the MiSeq reagent kit v3 150 cycles (Illumina, San Diego, CA, USA; Cat. #MS-102-3001). The reads were subjected to *de novo* assembly using Andrew and Aaron’s Awesome Assembly pipeline (A5) and ORFs were predicted using rapid prokaryotic genome annotation (PROKKA). After assembly, a total of 49 contigs and 14 scaffolds were generated to *L. monocytogenes* serovar 1/2a, while overall 78 contigs and 28 scaffolds were generated to serovar 4b.

Sequence assembly yielded a 2,990,228 bp *L. monocytogenes* serovar 1/2a complete genome with a G+C content of 37.8% and longest scaffold size of 1,477,456 bp, with an *N*_50_ of 509,790 bp and raw coverage of 236×. At the same time, for *L. monocytogenes* serovar 4b, sequence assembly yielded a 3,001,292 bp complete genome with a G+C content of 37.8% and longest scaffold size of 481,612 bp, with an *N*_50_ of 308,327 bp and raw coverage of 430×.

### Nucleotide sequence accession numbers.

These whole-genome shotgun projects have been deposited in GenBank under the accession no. LKHO00000000 and LKCY00000000 for serovars 1/2a and 4b, respectively. The versions described in this paper are the first versions, LKHO00000000 (serovar 1/2a) and LKCY00000000 (serovar 4b).
